# Fracture prevention with vitamin D supplementation: considering the inconsistent results

**DOI:** 10.1186/1471-2474-8-26

**Published:** 2007-03-09

**Authors:** Gerbrand J Izaks

**Affiliations:** 1 Department of General Internal Medicine and Geriatrics, University Medical Centre Groningen, P.O. Box 30.001, 9700 RB Groningen, The Netherlands

## Abstract

**Background:**

A meta-analysis found that high dose vitamin D, different from low dose, decreased fracture risk by 23% for any nonvertebral fracture and by 26% for hip fracture. Unfortunately, however, this effect was not confirmed by recent trials. The aim of this paper is to explore if this inconsistency can be attributed to publication bias or heterogeneity of the trials.

**Methods:**

The meta-analysis was extended with recent randomised controlled trials (RCTs) that were identified by a systematic review. Risk ratios (RR) and 95% confidence intervals (CI) were calculated from raw data. A funnel plot was used to explore the possibility of publication bias. Forest plots were used to investigate if vitamin D dose, concurrent use of calcium and target population were sources of heterogeneity. Linear regression analysis of log RR on adherence rate and achieved vitamin D level was used to study whether these variables were associated with fracture risk.

**Results:**

A total of eleven trials was included: seven RCTs from the meta-analysis and four recently published. For any nonvertebral fracture, the funnel plot was asymmetrical because two small RCTs showed a large positive effect. This was not found for hip fracture. As reported in the meta-analysis, low dose vitamin D (<400 IU daily) was not effective. In contrast to the meta-analysis, however, the effect of high dose vitamin D (≥700 IU daily) seemed to be dependent on target population. For any nonvertebral fracture, the pooled RR was 0.80 (95% CI, 0.70–0.90) in institutionalised persons, and 0.88 (95% CI, 0.75–1.04) in the general population; for hip fracture, pooled RR 0.72 (95% CI, 0.59 to 0.88) and 1.04 (95% CI, 0.72–1.50), respectively. Other sources of heterogeneity were not clearly found. In the meta-analysis, pooled RRs were mainly based on small trials that showed a large effect or trials in institutionalised persons.

**Conclusion:**

It is likely that the inconsistency between the meta-analysis and the recent trials is, at least partially, due to publication bias and differences in target population. High dose vitamin D may be effective in institutionalised persons but probably is not effective in the general population.

## Background

Controversy persists whether low-trauma or osteoporotic fractures can be prevented with vitamin D supplementation. In 2005, the divergent findings were addressed in a meta-analysis and the results were hopeful [1]. Although an oral vitamin D dose of 400 IU daily was not sufficient for fracture prevention, fracture risk was reduced by 23 to 26 percent if vitamin D supplementation was given in a daily dose of 700 to 800 IU. However, the positive effect of high dose vitamin D supplementation was not confirmed in more recent trials and Cochrane reviewers also came to a different conclusion [2-4]. Therefore, we must question why the results are inconsistent. In general, publication bias of the meta-analysis of 2005 or heterogeneity of the trials, for example due to differences in target population or adherence rate, are likely explanations. The aim of this paper is to investigate which of these factors may play a role.

## Methods

I performed a systematic review of the literature using MEDLINE (PubMed) to identify randomised controlled trials (RCTs) on fracture prevention with vitamin D supplementation that were published after the meta-analysis of 2005. "Cholecalciferol", "ergocalciferol", "25-hydroxyvitamin D 2", "vitamin D", "fracture" and "fall" were used as search terms (Medical Subject Heading (MeSH) and text word) and the search was limited to randomised controlled trials with publication date January 1, 2005 or later. Furthermore, I checked the ongoing trials described in the Cochrane review [4]. As a next step, I applied the same criteria for eligibility as was done in the meta-analysis [1]. A trial was included if it was a double-blind RCT that studied oral vitamin D supplementation (cholecalciferol or ergocalciferol) with a minimum follow-up of one year and if there was more than a total of one fracture in the trial. Measurement of 25-hydroxyvitamin D levels during follow-up was not required because it would lead to exclusion of two of the largest trials [2,5].

The raw data of all RCTs were entered in Review Manager (RevMan) 4.2.6 [6]to calculate risk ratios (RR) and 95 percent confidence intervals (CI), using a fixed effects model, and to draw a funnel plot.

To study possible sources of heterogeneity, risk ratios are presented according to target population (institutionalised persons, general population), vitamin D dose (low dose, 400 IU daily; high dose, ≥ 700 IU daily), and concurrent use of calcium supplements (no, yes). In addition, linear regression analysis of log RR on adherence rate, achieved vitamin D level, age and absolute fracture risk was carried out to study the influence of these variables on fracture risk. This was done in SPSS 12.0.1 for Windows (SPSS Inc, Chicago, Illinois, USA).

## Results

### Non-vertebral fractures

**Figure 1:**
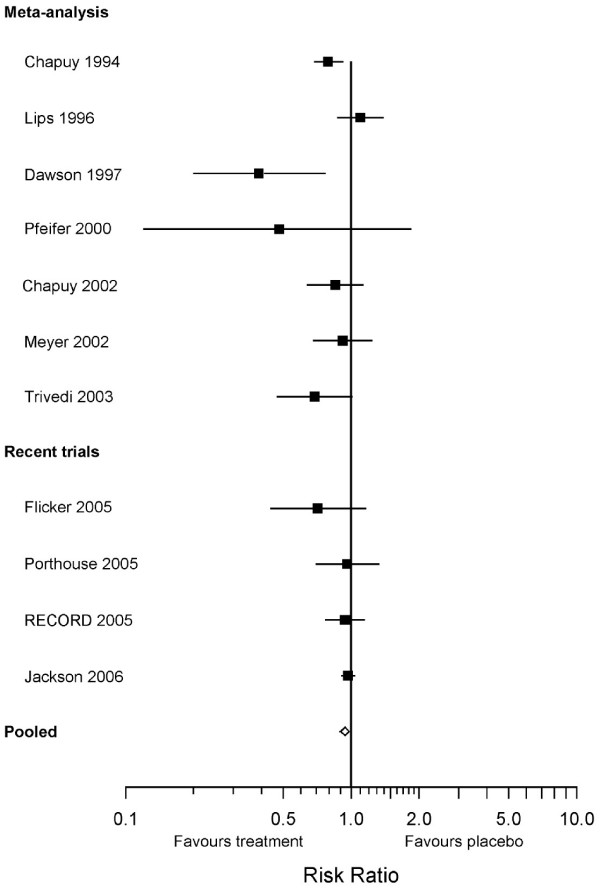
**Risk ratio for any nonvertebral fracture in a comparison of vitamin D supplementation (treatment) with placebo**. The trials are ordered according to year of publication. Bars represent 95 percent confidence intervals. Pooled risk ratio, 0.94 (95 percent confidence interval, 0.89 to 0.98).

Eleven RCTs reported on the risk of any nonvertebral fracture. Seven trials were included in the meta-analysis of 2005 [7-13], and four were published recently [2,3,5,14]. Two early RCTs showed a statistically significant decrease of fracture risk in the treatment group (figure 1) [7,9]. One RCT showed a decrease of fracture risk that was of borderline statistical significance [13]. Eight RCTs did not show a positive effect of vitamin D supplementation 2005 [2,3,5,8,10-12,14].

**Figure 2:**
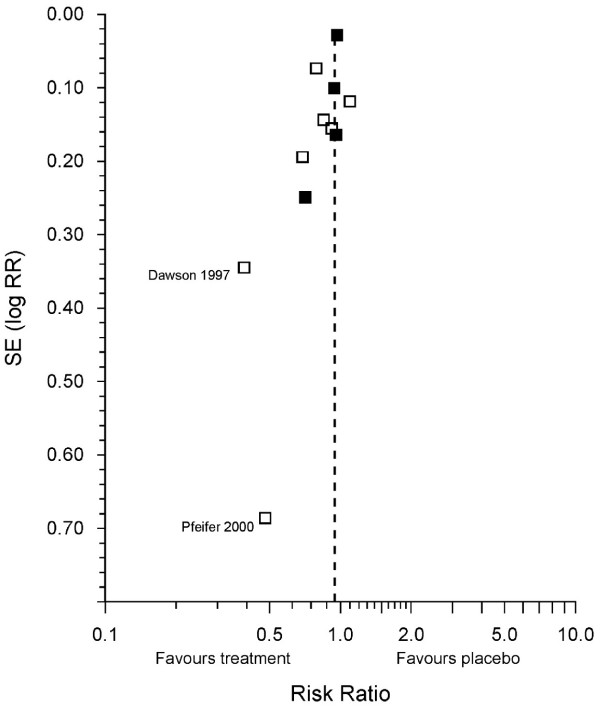
**Funnel plot of randomised controlled trials on the prevention of any nonvertebral fracture with vitamin D supplementation**. Open symbols: trials included in the meta-analysis. Solid symbols: recent trials. Dashed line: pooled risk ratio (RR, 0.94; 95 percent confidence interval, 0.89 to 0.98).

When a funnel plot is drawn, two RCTs clearly attract attention (figure 2). These trials were relatively small but showed the largest decrease of fracture risk in the treatment group. The Dawson trial included 389 persons and the Pfeifer trial 137 persons [9,10]. The risk ratios were 0.39 (95% CI, 0.20 to 0.77) and 0.48 (95% CI, 0.12 to 1.84), respectively. Both trials were included in the meta-analysis of 2005. The other trials are evenly distributed around the pooled risk ratio that is based on the data of the eleven RCTs (RR 0.94; 95 percent CI, 0.89 to 0.98).

**Figure 3:**
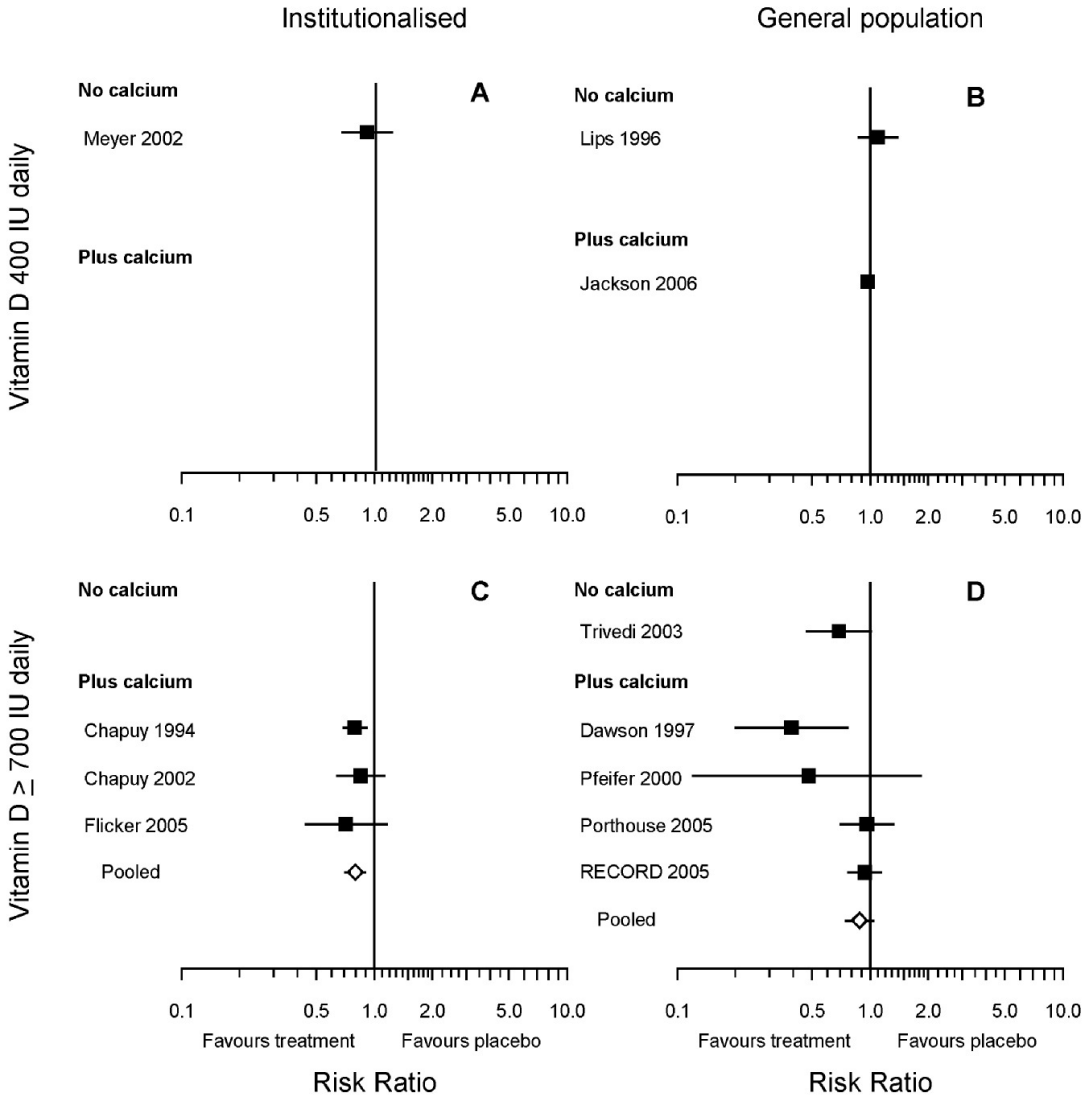
**Risk ratios for any nonvertebral fracture according to target population, vitamin D dose and concurrent use of calcium**. Bars represent 95 percent confidence intervals.

Publication bias, chance and hetereogeneity of the RCTs are the main factors to explain the asymmetry of the funnel plot. Heterogeneity may be due to differences in target population, vitamin D dose, and concurrent use of calcium supplements. Therefore, as a next step, forest plots were drawn according to these possible sources of heterogeneity (figures 3A–D).

Low dose vitamin D supplementation (400 IU daily) did not decrease fracture risk in any of the trials (figures 3A–B). The effect of high dose vitamin D supplementation (≥ 700 IU daily) was dependent on the target population. A consistent decrease of fracture risk was seen in institutionalised persons who took high dose vitamin D supplementation combined with extra calcium (600 to 1200 mg daily) (figure 3C) although it must be recognised that two of the three trials were performed by the same authors [7,11]. The pooled risk ratio in the treatment group was 0.80 (95 percent CI, 0.70 to 0.90) in institutionalised persons.

Four RCTs that used high dose vitamin D supplementation in combination with extra calcium (500 to 1200 mg daily) were performed in the general population (figure 3D). Here, again, the Dawson and Pfeifer trial attract attention. Although these small trials found a large decrease of fracture risk, two recent large trials found no effect. The pooled risk ratio in the treatment group was 0.88 (95% CI, 0.75 to 1.04) in the general population.

**Figure 4:**
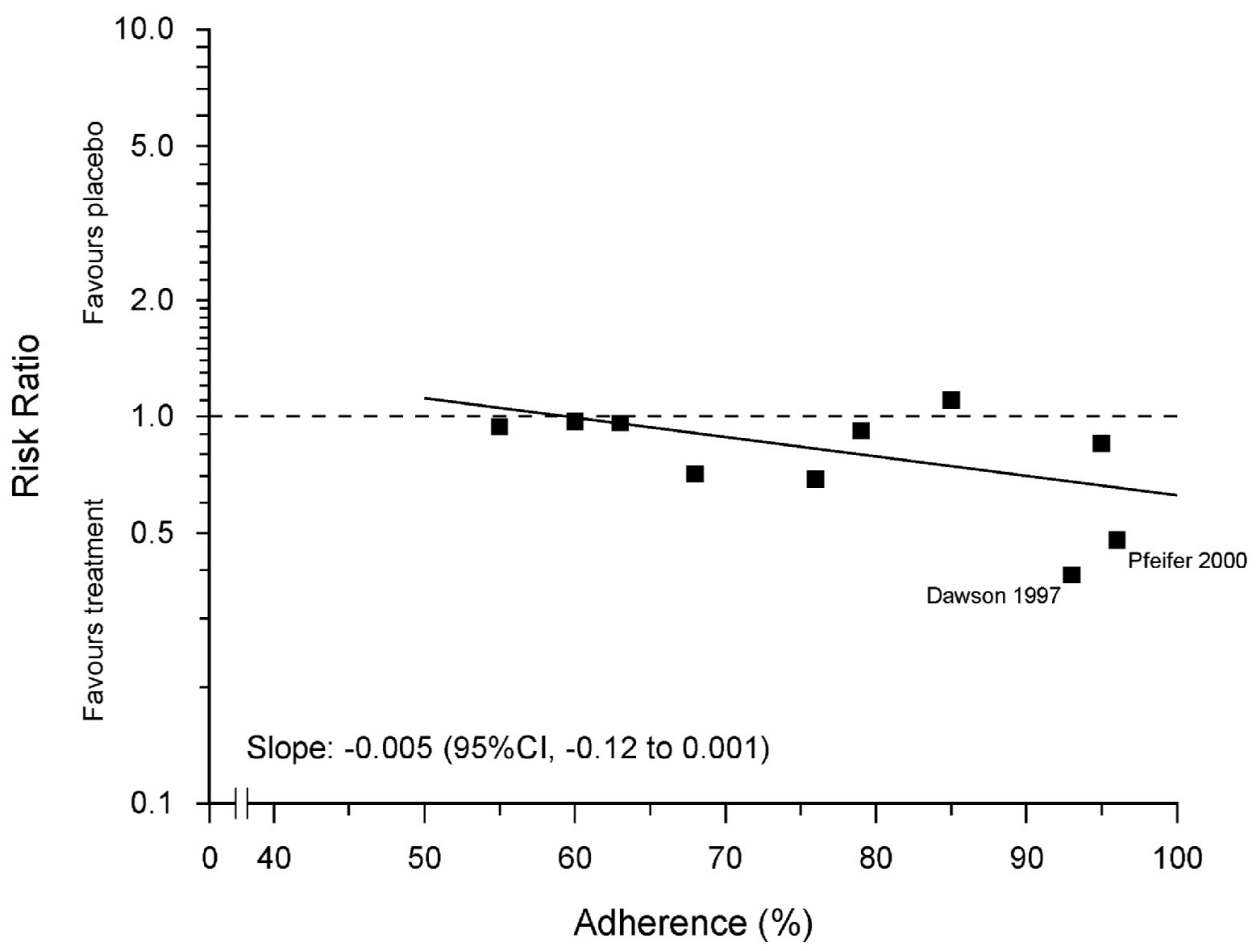
**Risk ratios for any nonvertebral fracture according to adherence to the trial medication**. Slope: slope of the regression line. 95%CI: 95 percent confidence interval. Pearson correlation coefficient: -0.56 (p = 0.10).

**Figure 5:**
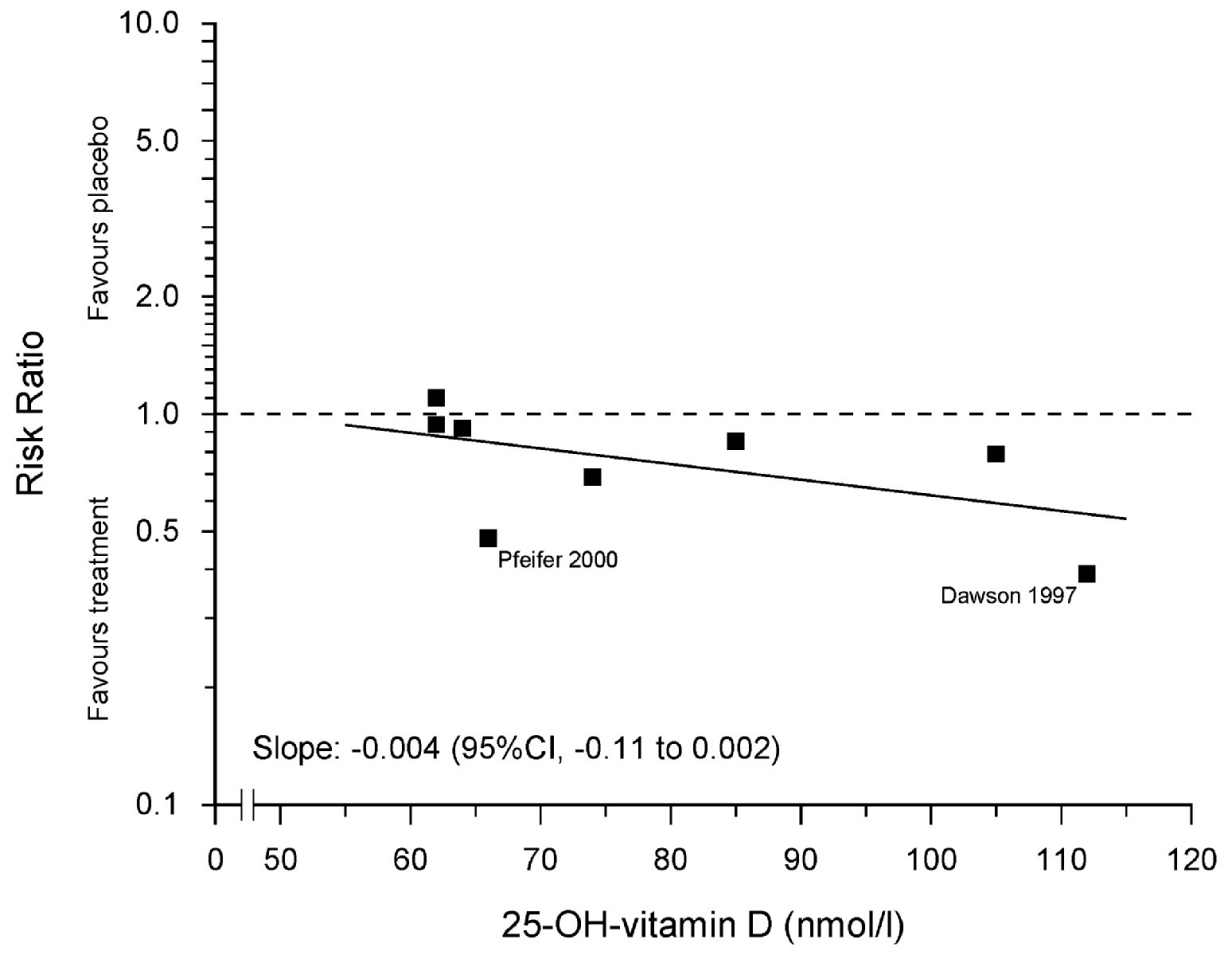
**Risk ratios for any nonvertebral fracture according to achieved vitamin D level**. Slope: slope of the regression line. 95%CI: 95 percent confidence interval. Pearson correlation coefficient: -0.54 (p = 0.17). Multiply values by 0.40 to convert 25-OH-vitamin D to ng/ml.

Most authors did not clearly describe their definition of adherence to the trial medication nor the method by which it was assessed. The reported adherence rates varied between 55 and 95 percent. The correlation between adherence and risk ratio was not statistically significant nor was the slope of the regression line statistically significant different from zero (figure 4). Similar results were found for the relation between risk ratio and achieved vitamin D concentration (figure 5), age and absolute fracture risk (data not shown).

### Hip fractures

Data on hip fracture were reported in eight RCTs [2,3,5,7,8,11-13]. Here also, the RCTs showed divergent results but there was no clear difference between RCTs included in the meta-analysis of 2005 and the recent RCTs, and the funnel plot was symmetrical (data not shown). No RCT showed a positive effect of low dose vitamin D [5,8,12]. High dose vitamin D seemed to be effective in institutionalised persons (two trials; RR 0.72; 95 percent CI, 0.59 to 0.88) [7,11] but not in the general population (two trials; RR 1.04; 95 percent CI, 0.72 to 1.50)[2,3].

## Discussion

First of all, it must be said that the number of RCTs on fracture prevention with vitamin D is too small for far reaching conclusions. The funnel plot may be misleading and the assessment of heterogeneity may be inadequate. Nevertheless, some cautious conclusions can be drawn.

Low dose vitamin D was not effective in any trial. This was also concluded by the authors of the meta-analysis of 2005. However, the effectiveness of high dose vitamin D seems to be dependent on the target population. High dose vitamin D lowered fracture risk by about 20 percent in institutionalised persons but did not result in a statistically significant decrease in fracture risk in the general population.

It is plausible that the effect of vitamin D supplementation in institutionalised persons is larger than in the general population. As institutionalised persons are exposed to sunlight less often than persons from the general population, it can be assumed that without supplementation the vitamin D levels are lower in institutionalised persons. Accordingly, it may be expected that supplementation has a larger effect in this group. Thus, if we estimate the effect of vitamin D supplementation on fracture risk, we must distinguish between institutionalised persons and persons from the general population.

In the meta-analysis, ìt was estimated that high dose vitamin D lowered the risk of any nonvertebral fracture by 23 percent. The estimate was based on the pooled results of five RCTs [7,9-11,13]. Two of the trials were performed in institutionalised persons and three in the general population. As stated above, the effect of vitamin D may be larger in institutionalised persons. By pooling the data from institutionalised persons and the general population, the meta-analysis probably overestimated the effect of vitamin D supplementation.

Furthermore, two of the three trials in the general population that were included in the meta-analysis were small but showed a large positive effect [9,10]. As illustrated by the asymmetry of the funnel plot, there were no small trials with a negative effect. This may be due to publication bias as publication bias is more likely to affect small rather than large trials [15]. Obviously, the asymmetry of the funnel plot also may be due to chance because the number of included trials is small. However, apart from the underlying mechanism, inclusion of the small studies also may have led to overestimation of the effect of high dose vitamin D in the meta-analysis.

Apart from vitamin D dose and target population, adherence rate to the trial medication and achieved vitamin D concentration are often mentioned as possible sources of heterogeneity. Bearing in mind its limitations, the present analysis did not clearly show an association. Some might argue that an association is suggested by the negative slopes of the regression lines but this also seems in large part due to the small Dawson and Pfeifer trials. Naturally, it may be mentioned that the effect in the Dawson trial was large precisely because the adherence rate and the achieved vitamin D level were high but studies with similar adherence rates or vitamin D levels did not show such large effects.

## Conclusion

It is likely that the inconsistency between the meta-analysis and the recent trials is, at least partially, due to publication bias and differences in target population. High dose vitamin D may be effective in institutionalised persons but probably is not effective in the general population.

## Competing interests

The author(s) declare that they have no competing interests.

## Authors' contributions

GI was the sole author of this article.

## Pre-publication history

The pre-publication history for this paper can be accessed here:



## References

[B1] Bischoff-Ferrari HA, Willett WC, Wong JB, Giovannucci E, Dietrich T, Dawson-Hughes B (2005). Fracture prevention with vitamin D supplementation: a meta-analysis of randomized controlled trials. JAMA.

[B2] Porthouse J, Cockayne S, King C, Saxon L, Steele E, Aspray T, Baverstock M, Birks Y, Dumville J, Francis R, Iglesias C, Puffer S, Sutcliffe A, Watt I, Torgerson DJ (2005). Randomised controlled trial of calcium and supplementation with cholecalciferol (vitamin D3) for prevention of fractures in primary care. BMJ.

[B3] Grant AM, Avenell A, Campbell MK, McDonald AM, MacLennan GS, McPherson GC, Anderson FH, Cooper C, Francis RM, Donaldson C, Gillespie WJ, Robinson CM, Torgerson DJ, Wallace WA (2005). Oral vitamin D3 and calcium for secondary prevention of low-trauma fractures in elderly people (Randomised Evaluation of Calcium Or vitamin D, RECORD): a randomised placebo-controlled trial. Lancet.

[B4] Avenell A, Gillespie WJ, Gillespie LD, O'Connell DL (2005). Vitamin D and vitamin D analogues for preventing fractures associated with involutional and post-menopausal osteoporosis. Cochrane Database Syst Rev.

[B5] Jackson RD, LaCroix AZ, Gass M, Wallace RB, Robbins J, Lewis CE, Bassford T, Beresford SA, Black HR, Blanchette P, Bonds DE, Brunner RL, Brzyski RG, Caan B, Cauley JA, Chlebowski RT, Cummings SR, Granek I, Hays J, Heiss G, Hendrix SL, Howard BV, Hsia J, Hubbell FA, Johnson KC, Judd H, Kotchen JM, Kuller LH, Langer RD, Lasser NL, Limacher MC, Ludlam S, Manson JE, Margolis KL, McGowan J, Ockene JK, O'Sullivan MJ, Phillips L, Prentice RL, Sarto GE, Stefanick ML, Van Horn L, Wactawski-Wende J, Whitlock E, Anderson GL, Assaf AR, Barad D (2006). Calcium plus vitamin D supplementation and the risk of fractures. N Engl J Med.

[B6] (2007). The Information Management System (IMS) http://www.cc-ims.net/.

[B7] Chapuy MC, Arlot ME, Delmas PD, Meunier PJ (1994). Effect of calcium and cholecalciferol treatment for three years on hip fractures in elderly women. BMJ.

[B8] Lips P, Graafmans WC, Ooms ME, Bezemer PD, Bouter LM (1996). Vitamin D supplementation and fracture incidence in elderly persons. A randomized, placebo-controlled clinical trial. Ann Intern Med.

[B9] Dawson-Hughes B, Harris SS, Krall EA, Dallal GE (1997). Effect of calcium and vitamin D supplementation on bone density in men and women 65 years of age or older. N Engl J Med.

[B10] Pfeifer M, Begerow B, Minne HW, Abrams C, Nachtigall D, Hansen C (2000). Effects of a short-term vitamin D and calcium supplementation on body sway and secondary hyperparathyroidism in elderly women. J Bone Miner Res.

[B11] Chapuy MC, Pamphile R, Paris E, Kempf C, Schlichting M, Arnaud S, Garnero P, Meunier PJ (2002). Combined calcium and vitamin D3 supplementation in elderly women: confirmation of reversal of secondary hyperparathyroidism and hip fracture risk: the Decalyos II study. Osteoporos Int.

[B12] Meyer HE, Smedshaug GB, Kvaavik E, Falch JA, Tverdal A, Pedersen JI (2002). Can vitamin D supplementation reduce the risk of fracture in the elderly? A randomized controlled trial. J Bone Miner Res.

[B13] Trivedi DP, Doll R, Khaw KT (2003). Effect of four monthly oral vitamin D3 (cholecalciferol) supplementation on fractures and mortality in men and women living in the community: randomised double blind controlled trial. BMJ.

[B14] Flicker L, MacInnis RJ, Stein MS, Scherer SC, Mead KE, Nowson CA, Thomas J, Lowndes C, Hopper JL, Wark JD (2005). Should older people in residential care receive vitamin D to prevent falls? Results of a randomized trial. J Am Geriatr Soc.

[B15] Sterne JAC, Egger M, Davey Smith G, Egger M, Davey Smith G and Altman DG (2001). Investigating and dealing with publication and other biases. Systematic reviews in health care: meta-analysis in context.

